# Impact of preoperative fecal short chain fatty acids on postoperative infectious complications in esophageal cancer patients

**DOI:** 10.1186/s12876-020-01217-y

**Published:** 2020-03-16

**Authors:** Masaaki Motoori, Koji Tanaka, Keijiro Sugimura, Hiroshi Miyata, Takuro Saito, Yasuhiro Miyazaki, Kazumasa Fujitani, Yukiko Kado, Takashi Asahara, Masahiko Yano

**Affiliations:** 1 Department of Surgery, Osaka International Cancer Institute, 3-1-69 Otemae, Osaka, Chuo-ku 541-8567 Japan; 2 Department of Surgery, Osaka General Medical Center, 3-1-56 Bandai-higashi, Osaka, 558-8558 Japan; 3 Department of Gastroenterological Surgery, Graduate School of Medicine, Osaka University, 2-2 Yamadaoka Suita, Osaka, 565-0875 Japan; 4 Yakult Central Institute, 1796 Yaho, Kunitachi, Tokyo 186-8650 Japan

**Keywords:** Esophageal cancer, Esophagectomy, Postoperative complications, Intestinal environment, Organic acids

## Abstract

**Background:**

The intestinal epithelial barrier allows absorption of dietary nutrients and prevents passage of pathogens and toxins into the body. Severe insults have a negative impact on the intestinal environment, which may decrease intestinal barrier function and cause bacterial translocation. Bacterial translocation, which can cause infectious complications, is defined as the passage of microbes from the gastrointestinal tract across the mucosal barrier to extraintestinal sites. The aim of this study was to investigate the correlation between concentrations of preoperative fecal organic acids and the occurrence of postoperative infectious complications in patients with esophageal cancer.

**Methods:**

Fifty-five patients with esophageal cancer who underwent esophagectomy were enrolled in this study. Perioperative synbiotics were administered to all patients. Perioperative clinical characteristics and concentrations of preoperative fecal organic acids were compared between patients with and without postoperative infectious complications.

**Results:**

Postoperative infectious complications occurred in 10 patients. In patients with complications, the concentrations of acetic acid and propionic acid were significantly lower than in patients without complications (*p* = 0.044 and 0.032, respectively). The concentration of butyric acid was nonsignificantly lower in patients with complications, while the concentration of lactic acid was nonsignificantly higher. The calculated gap between the concentrations of fecal acetic acid plus propionic acid plus butyric acid minus lactic acid was significantly lower in patients with complications. Multivariate analysis revealed that a low gap between acetic acid plus propionic acid plus butyric acid minus lactic acid was an independent risk factor for postoperative infectious complications (*p* = 0.027).

**Conclusions:**

Preoperative fecal concentrations of organic acids had a clinically important impact on the occurrence of postoperative infectious complications in patients with esophageal cancer. To reduce postoperative infectious complications, it may be useful to modulate the intestinal environment and maintain concentrations of fecal organic acids before surgery.

## Background

Subtotal esophagectomy for esophageal cancer is one of the most invasive gastrointestinal surgeries and is associated with high morbidity and mortality rates, though there have been recent advances in surgical techniques and perioperative management [1, 2]. The intestinal tract is the largest immune organ in the human body. The intestinal epithelial barrier allows absorption of dietary nutrients and prevents passage of pathogens and toxins into the body. Severe insults have a negative impact on the intestinal environment, which may decrease intestinal barrier function and cause bacterial translocation. We previously reported that esophagectomy in patients with esophageal cancer disturbed the intestinal microbiota and decreased the concentrations of organic acids, while the administration of perioperative synbiotics significantly shortened the duration of the postoperative systemic inflammatory response syndrome and nonsignificantly reduced postoperative infectious complications by maintaining the intestinal microbiota and the concentrations of organic acids [3].

The fermentation of carbohydrates by the intestinal microbiota produces organic acids. Among them, short chain fatty acids (SCFAs) play a variety of important roles in maintaining intestinal mucosal barrier function and preventing bacterial translocation [4]. Bacterial translocation, which can cause infectious complications, is the passage of microbes or their products from the gastrointestinal tract across the mucosal barrier to extraintestinal sites, such as the mesenteric lymph nodes, liver, or bloodstream. Several investigators demonstrated that severe insults, such as major surgery and the severe systemic inflammatory response syndrome, disrupt the intestinal environment, in particular disturbing the intestinal microbiota and decreasing the concentrations of organic acids [3, 5, 6]. However, there is only one study concerning the association between the preoperative intestinal environment and postoperative infectious complications. Yokoyama et al. reported that low preoperative fecal concentrations of SCFAs were significantly associated with the occurrence of postoperative infectious complications in patients undergoing major hepatectomy with extrahepatic bile duct resection [7].

The aim of this study was to investigate the correlation between the preoperative fecal concentrations of organic acids and the occurrence of postoperative infectious complications in patients with esophageal cancer undergoing esophagectomy.

## Methods

### Patients

This study included 55 patients with thoracic esophageal cancer who underwent transthoracic esophagectomy at Osaka International Cancer Institute. These patients were included in our previous studies [3, 8]. All patients received gastrointestinal fiberscopy and computed tomography scans of the neck, chest, and upper abdomen for tumor staging according to the TNM classification (7th edition) [9]. Patients underwent transthoracic subtotal esophagectomy with two- or three-field lymphadenectomy and reconstruction using the stomach or jejunum. An enteral feeding tube was inserted through the stomach or jejunum during the operation. Synbiotics were administered to all patients from 5 or more days before surgery to 21 days after surgery. The administered probiotics were Yakult BL Seichoyaku (Yakult Honsha, Tokyo), containing 1 × 10^8^ live *Bifidobacterium breve* strain Yakult and 1 × 10^8^ live *Lactobacillus casei* strain Shirota/g, and the prebiotics were galacto-oligosaccharides (Oligomate S-HP; Yakult Honsha). Before surgery, Yakult BL Seichoyaku (3 g/day) and galacto-oligosaccharides (15 g/day) were administered orally, and after surgery, Yakult BL Seichoyaku (2 g/day) and galacto-oligosaccharides (10 g/day) were administered through an enteral feeding tube or orally. Enteral feeding using a polymeric formula was initiated on postoperative day 1. Postoperative complications were assessed according to the Clavien-Dindo classification [10]. In this study, postoperative infectious complications were defined as those of grade II or higher.

### Fecal bacteriologic and organic acid analysis

Fecal samples were collected 1 day before surgery. For measurement of the number of microbiota, fecal samples were placed into a tube containing RNAlater (Ambion, Austin, TX) and stored at − 20 °C until use. Total RNA was extracted from feces and the gut microbiota composition was examined using 16S or 23S ribosomal RNA–targeted reverse transcription–quantitative PCR using the Yakult Intestinal Flora-SCAN as described previously [11, 12]. For measurement of the concentrations of organic acids, fecal samples were placed into a vacant tube and stored at − 20 °C until use. Feces were homogenized in perchloric acid and the concentrations of organic acids were measured using a Waters high-performance liquid chromatography system (Waters 432 Conductive Detector; Waters, Milford, MA) and a Shodex RSpak KC-811 column (Showa Denko, Tokyo, Japan) [13].

### Statistical analysis

Statistical analyses were performed using JMP13 (SAS Institute) software. The relationships between postoperative infectious complications and clinical factors, numbers of fecal bacteria, or concentrations of fecal organic acids were evaluated by the chi-square test or the Mann-Whitney U test. Multivariate analysis of infectious complications was performed using multiple logistic regression. Differences were significant at *p* < 0.05.

The study protocol was approved by the Human Ethics Review Committees of Osaka International Cancer Institute and registered in the University Hospital Medical Information Network (http://www.umin.ac.jp; registration number ID UMIN000004704 and UMIN000006875). A signed consent form was obtained from each patient.

## Results

### Patient characteristics and surgical outcome

The clinical characteristics of the patients are presented in Table 1. The clinical disease stages were Stage I in 16 patients, Stage II in 15 patients, Stage III in 20 patients, and Stage IV (due to supraclavicular lymph node metastasis) in four patients. Neoadjuvant chemotherapy (NAC) or chemoradiotherapy was performed in 36 patients.

**Table 1:** Patient characteristics

Preoperative variables		
Gender	male/female	46/9
Age	median (range)	64 (37–76)
Tumor location	upper/middle/lower	6/30/19
Preoperative therapy	no/yes	19/36
cT factor	T1/T2/T3/T4	12/15/26/2
cN factor	N0/N1/N2/N3	25/19/10/1
cM factor	M0/M1	51/4
cStage	I/II/III/IV	16/15/20/4
Intraoperative variables		
Field of lymph node dissection	two-field/three-field	15/40
Organ used in reconstruction	stomach/jejunum	54/1
Operation time (min)	median (range)	558 (411–755)
Blood loss (ml)	median (range)	945 (370–3525)

Postoperative infectious complications occurred in 10 patients (18%), including pulmonary complications in seven, anastomotic leakage in one, pyothorax in one, and wound infection in two. There was no perioperative mortality.

Table 2 shows the association between preoperative and intraoperative factors and postoperative infectious complications. Postoperative infectious complications were more common in the elderly, but not significantly so (*p* = 0.073).

**Table 2:** Correlation between preoperative or intraoperative factors and postoperative infectious complications

		Complication (−)	Complication (+)	*p* value
		(*n* = 45)	(*n* = 10)	
Gender	male/female	37/8	9/1	0.55
Age		61.4 ± 8.7	66.8 ± 4.9	0.073
Tumor location	upper, middle / lower	30/15	6/4	0.69
Preoperative therapy	no/yes	17/28	2/8	0.28
cStage	I–II/III–IV	19/26	5/5	0.65
BMI (kg/m^2^)		21.8 ± 2.9	21.0 ± 1.6	0.39
Operation time (min)		546 ± 78	589 ± 78	0.15
Blood loss (ml)		1045 ± 517	988 ± 413	0.98

**Fig. 1:**
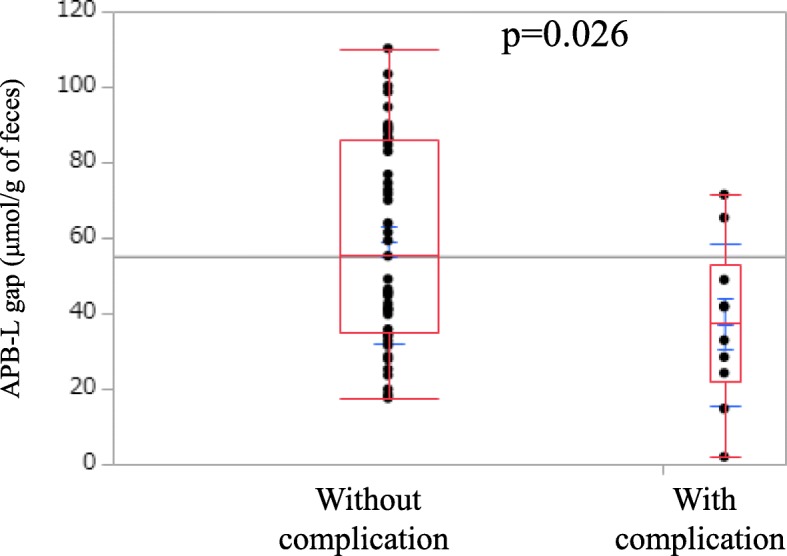
APB-L gap in patients with and without postoperative infectious complications. The box shows the interquartile range. The top and bottom bars represent the maximum and minimum values, respectively

Table 3 shows the association between the number of representative fecal obligate anaerobes, administered probiotics, or fecal organic acid concentrations, and postoperative infectious complications. There was no significant difference in the number of representative fecal obligate anaerobes between patients with and without postoperative complications. The number of *Bifidobacterium breve* strain Yakult was nonsignificantly lower in patients with complications than in those without. By contrast, the concentrations of acetic acid and propionic acid were significantly lower in patients with complications (*p* = 0.044 and 0.032, respectively). The concentration of butyric acid was nonsignificantly lower and the concentration of lactic acid was nonsignificantly higher in patients with complications. The concentration gap between acetic acid plus propionic acid plus butyric acid minus lactic acid (APB-L gap) was significantly lower in patients with complications than in those without. (46.3 ± 25.1 μmol/g of feces vs. 75.2 ± 34.3 μmol/g of feces, respectively, *p* = 0.026), as shown in Fig. 1. There was no significant correlation between fecal organic acid concentrations and the number of representative fecal obligate anaerobes (data not shown).

**Table 3:** Correlation between fecal microbiota or organic acids and postoperative infectious complications

	Complication (−)	Complication (+)	*p* value
	(*n* = 45)	(*n* = 10)	
Fecal microbiota (log_10_ cells/g of feces)			
*Clost. coccoides group*	9.46 ± 0.56	8.65 ± 2.35	0.77
*Clost. leptam subgroup*	9.22 ± 1.38	8.51 ± 2.36	0.3
*Bacteroides fragilis group*	9.29 ± 1.70	8.71 ± 2.00	0.52
*Bifidobacterium*	9.80 ± 1.12	9.79 ± 0.67	0.35
*Atopobium cluster*	9.21 ± 0.74	8.80 ± 1.2	0.23
*Prevotella*	6.58 ± 2.17	5.94 ± 2.38	0.48
*Total Lactobacillus*	7.56 ± 0.16	7.43 ± 1.18	0.50
*Bifidobacterium breve* strain Yakult	8.03 ± 1.23	6.76 ± 2.32	0.11
*Lactobacillus casei* strain Shirota	6.77 ± 2.17	5.93 ± 2.38	0.27
Fecal organic acid (μmol/g of feces)			
Total organic acids	80.9 ± 29.8	56.9 ± 37.0	0.11
Acetic acid	56.0 ± 25.8	36.7 ± 19.6	0.044
Propionic acid	16.0 ± 10.2	9.2 ± 4.8	0.032
Butyric acid	4.5 ± 3.9	2.6 ± 2.2	0.19
Lactic acid	1.3 ± 2.6	2.3 ± 3.4	0.12
Valeric acid	0.88 ± 0.59	0.74 ± 0.28	0.39
Iso-valeric acid	1.34 ± 0.92	0.90 ± 0.32	0.11
Formic acid	0.97 ± 1.5	1.2 ± 1.6	0.57
Succinic acid	2.3 ± 6.0	4.7 ± 8.6	0.67

Table 4 shows the multivariate analysis for postoperative infectious complications. The APB-L gap cutoff value was determined using receiver operating characteristics curve analysis. Among factors with *p* < 0.2 in the univariate analysis, the APB-L gap was the only factor significantly associated with postoperative infectious complications (*p* = 0.027).

**Table 4:** Multivariate analysis of postoperative infectious complications

		Odds ratio (95% CI)	*p* value
Age	≥70 (vs. ≤69)	1.24 (0.28–5.80)	0.77
Operation time (min)	≥550 (vs. < 550)	3.47 (0.79–18.8)	0.11
APB-L gap^a^ (μmol/g of feces)	≤85 (vs. > 85)	8.04 (1.24–160.34)	0.027

^a^: gap between acetic acid plus propionic acid plus butyric acid minus lactic acid

## Discussion

Patients with esophageal cancer who undergo esophagectomy have various risk factors that can negatively impact the intestinal environment, such as malnutrition due to cancer stenosis, preoperative therapy, surgical stress, use of antibiotics, postoperative fasting, and parenteral nutrition. If the preoperative intestinal environment is maintained, postoperative infectious complications may be reduced. This study revealed that a low APB-L gap was an independent risk factor for postoperative infectious complications. Preoperative concentrations of acetic acid, propionic acid, and butyric acid, which make up the majority of SCFAs (> 95%) [14], had a clinically important impact. These results are consistent with those of the previous study by Yokoyama et al. [7]. In their study of patients undergoing major hepatectomy with extrahepatic bile duct resection, the preoperative fecal concentrations of acetic acid and butyric acid were significantly lower and the concentration of lactic acid was nonsignificantly lower in patients who developed postoperative infectious complications, and the acetic acid plus butyric acid minus lactic acid gap was an independent risk factor for postoperative infectious complications. Lactic acid is digested by obligate anaerobes to produce SCFAs [15]. Yokoyama et al. speculated that a low acetic acid plus butyric acid minus lactic acid gap may reflect an impaired intestinal environment and thus lead to postoperative infectious complications [7].

SCFAs play various roles in maintaining the intestinal environment, and contribute to the prevention of bacterial translocation. SCFAs stimulate the proliferation and differentiation of intestinal epithelial cells and the secretion of mucus by goblet cells [16, 17]. They also maintain the acidity of the intestine and suppress the growth of harmful bacteria. Acetic acid, which is the most common organic acid, has an antimicrobial effect on harmful bacteria [18] and promotes the defensive functions of host epithelial cells [19]. Furthermore, Hsieh et al. reported that acetic acid strengthened tight junctions in Caco-2 monolayers [20]. Ohata et al. reported that propionic acid and butyric acid induced tight junction permeability in Caco-2 monolayer cells. Butyric acid is the major source of colonic epithelial cells, and induces the differentiation of regulatory T cells and ameliorates chronic intestinal inflammation in mice [21]. Thus, maintaining the concentration of SCFAs prior to iatrogenic gastrointestinal tract damage is useful for preventing bacterial translocation.

Organic acids are mainly produced in the large intestine by obligate anaerobes. In this study, there was no significant difference between the two groups in the number of representative fecal obligate anaerobes, as shown in Table 3. This result is inconsistent with that of the prior study mentioned above [7]. We previously reported that in patients with advanced esophageal cancer, NAC altered the intestinal microbiota and concentrations of organic acids [8]. In this study, about two-thirds of patients received preoperative chemotherapy or preoperative chemoradiotherapy. We performed a subanalysis in only patients who received preoperative therapy, examining the correlation between either the preoperative number of representative fecal obligate anaerobes or the fecal concentrations of organic acids and the incidence of postoperative infectious complications. There was no significant difference in the number of representative fecal obligate anaerobes between the two groups, while the concentrations of acetic acid and propionic acid were significantly lower in patients with complications than in those without (*p* = 0.025 and 0.022, respectively, data not shown). The concentration of SCFAs is considered to reflect the function of the intestinal microbiota, which may be affected by preoperative therapy. Thus, the concentration of SCFAs but not the number of representative fecal obligate anaerobes was useful for predicting postoperative infectious complications in this study.

Acetic acid, which is the most common SCFA, is mainly generated by *Bifidobacterium* [19, 22]. In this study, the concentration of acetic acid was significantly lower in patients with complications than in those without. This may be because while the number of *Bifidobacterium* did not differ between the two groups, the number of *Bifidobacterium breve* strain Yakult, one of the administered probiotics, was nonsignificantly higher in patients without complications. Since *Bifidobacterium breve* strain Yakult can selectively metabolize galacto-oligosaccharide, it may increase the production of acetic acid [23]. In this study, although all patients received synbiotics, including *Bifidobacterium breve* strain Yakult, for at least 5 days before surgery, the number of preoperative fecal *Bifidobacterium breve* strain Yakult was nonsignificantly lower in the patients with complication than in those without complication. It is considered that different patients had different responses to synbiotics. The mechanism of this variation is unclear. Currently, we administer perioperative synbiotics to all patients with esophageal cancer scheduled to undergo esophagectomy. Therefore, if a patient has low concentrations of SCFAs after receiving synbiotics, more careful attention should be paid during the perioperative period to prevent postoperative infectious complications. If high-performance liquid chromatography and reagents are available, the concentrations of fecal SCFAs can be measured in a few hours, and the cost is about 2000 Japanese yen per sample. Measurement of the concentrations of fecal SCFAs, which is a useful indicator for the occurrence of postoperative infectious complications, should be incorporated into daily clinical practice in the future.

Our study has several limitations. First, the number of enrolled patients was small. Second, about two-thirds of patients received preoperative chemotherapy or chemoradiotherapy, both of which alter the intestinal environment, while about one-third of patients received no preoperative therapy. Data of fecal microbiota and organic acid concentrations before and after NAC, that is, before surgery were available for 25 of the 55 cases enrolled in this study. Synbiotics were administered to these 25 patients during NAC. The number of bacteria administered as probiotics increased significantly after NAC, while no difference was found in the number of other bacteria before and after NAC. The concentration of butyric acid was significantly decreased after NAC (*p* = 0.0007). On the other hand, data of perioperative fecal microbiota and organic acid concentrations (before the administration of synbiotics, 1 day before surgery and 1 and 3 weeks after surgery) were available in 30 of the 55 patients enrolled in this study. Eleven patients received preoperative therapy without synbiotics and remaining 19 patients did not receive preoperative therapy. The number of *Clostridium leptum subgroup* before the administration of synbiotics was significantly larger in patients with preoperative therapy than in those without (*p* = 0.047). No difference was observed in the number of Clostridium leptum subgroup at other time points, or in the number of other bacteria or organic acid concentrations between the two groups. In addition, the duration of synbiotics administration varied among individuals. This heterogeneity may have affected the results of our study. Third, there are no data concerning intestinal permeability, which correlates with the degree of bacterial translocation.

## Conclusion

Preoperative fecal concentrations of acetic acid and propionic acid were significantly lower in patients who developed postoperative infectious complications than in those who did not. To reduce postoperative infectious complications after esophagectomy in patients with esophageal cancer, it may be useful to modulate the intestinal environment and maintain concentrations of fecal SCFAs.

## Data Availability

The datasets used and/or analyzed during the current study are available from the corresponding author on reasonable request.

## Abbreviations

APB-L gap: The concentration gap between the acetic acid plus propionic acid plus butyric acid minus lactic acid
NAC: Neoadjuvant chemotherapy
SCFAs: Short chain fatty acids
